# Toward Quantitative *in vivo* Label-Free Tracking of Lipid Distribution in a Zebrafish Cancer Model

**DOI:** 10.3389/fcell.2021.675636

**Published:** 2021-07-01

**Authors:** Marco Andreana, Caterina Sturtzel, Clemens P. Spielvogel, Laszlo Papp, Rainer Leitgeb, Wolfgang Drexler, Martin Distel, Angelika Unterhuber

**Affiliations:** ^1^Center for Medical Physics and Biomedical Engineering, Medical University of Vienna, Vienna, Austria; ^2^Innovative Cancer Models, St. Anna Children's Cancer Research Institute, Vienna, Austria; ^3^Zebrafish Platform Austria for Preclinical Drug Screening (ZANDR), Vienna, Austria; ^4^Division of Nuclear Medicine, Department of Medical Imaging and Image-Guided Therapy, Medical University of Vienna, Vienna, Austria; ^5^Christian Doppler Laboratory for Applied Metabolomics, Medical University of Vienna, Vienna, Austria; ^6^Christian Doppler Laboratory OPTRAMED, Medical University of Vienna, Vienna, Austria

**Keywords:** zebrafish, cancer model, lipid metabolism, label-free microscopy, coherent anti-Stokes Raman scattering

## Abstract

Cancer cells often adapt their lipid metabolism to accommodate the increased fatty acid demand for membrane biogenesis and energy production. Upregulation of fatty acid uptake from the environment of cancer cells has also been reported as an alternative mechanism. To investigate the role of lipids in tumor onset and progression and to identify potential diagnostic biomarkers, lipids are ideally imaged directly within the intact tumor tissue in a label-free way. In this study, we investigated lipid accumulation and distribution in living zebrafish larvae developing a tumor by means of coherent anti-Stokes Raman scattering microscopy. Quantitative textural features based on radiomics revealed higher lipid accumulation in oncogene-expressing larvae compared to healthy ones. This high lipid accumulation could reflect an altered lipid metabolism in the hyperproliferating oncogene-expressing cells.

## 1. Introduction

Cancer is the second leading cause of death responsible for about 1 out of 6 deaths worldwide. According to the World Health Organization one defining feature of cancer is the rapid creation of abnormal cells that grow beyond their usual boundaries and then invade adjoining parts of the body and spread to other organs (WHO, 2010). These abnormal cells differ from their non-transformed counterparts in morphology, proliferative and migratory potential, metabolism, and cellular interactions. Metabolic adaptation of cancer cells, including lipid metabolism, has been identified as one of the important factors for tumor growth (Vander Heiden and DeBerardinis, 2017).

Lipids are organic molecules used by cells as an essential component of membranes compartmentalizing structures and organelles within the cell as well as separating the cell from other cells. Lipids are also used to store energy and to modify proteins (Munir et al., 2019). Due to their increased proliferation, cancer cells are in high demand of fatty acids for the synthesis of lipid membranes and to provide energy. This often induces alterations in lipid metabolism to satisfy the increased fatty acid demand and to allow for the survival of cancer cells. Alteration in lipid metabolism has become a hallmark in many solid tumors and has been recognized as an important rewriting phenomenon in tumor cells (Maan et al., 2018). Several studies have demonstrated that changes in lipid content could serve as a novel biomarker for cancer detection; furthermore, targeting lipid metabolism could be an innovative therapeutic anti-cancer strategy (Long et al., 2018; Maan et al., 2018). Therefore, studying lipid metabolism at cellular and subcellular levels will give new insights into carcinogenesis to instruct the development of targeted and efficient treatment of patients. As lipid metabolism is largely conserved among species ranging from plant (Nguyen et al., 2011), yeast (van Zutphen et al., 2014), *C. elegans* (Lapierre et al., 2011) to zebrafish (Anderson et al., 2011) and humans, animal models can be used to obtain relevant insights (Schlegel and Stainier, 2007). In preclinical cancer research, zebrafish (*Danio rerio*) has gained popularity over the last years and has been established as an *in vivo* cancer model offering many benefits. Zebrafish are small, easy, and cheap to house. Moreover, this small fresh water fish shares approximately 81% of human disease related genes (Howe et al., 2013). Nowadays, several zebrafish cancer models have been established, including leukemia, melanoma, neuroblastoma, liver, pancreatic, and testicular cancer (Mione and Trede, 2010). High fecundity, with females producing up to 300 embryos per week, makes such models especially suitable for large-scale drug screening. Most importantly, the transparent nature of zebrafish embryos provides a unique opportunity to investigate cancer cells and their microenvironment by optical microscopy. Indeed, by using transgenic fluorescent zebrafish reporter lines highlighting distinct cell populations or activity of signaling pathways by fluorescence or even by observing endogenous markers in wild-type zebrafish, optical microscopy can objectively and non-invasively identify, evaluate, and assess changes between healthy and pathological embryos. This offers even the possibility of longitudinal studies with follow up over several days (Lieschke and Currie, 2007; Keller, 2013; Abu-Siniyeh and Al-Zyoud, 2020).

Fluorescence microscopy based on single photon absorption is the most common approach used to track specific cell populations labeled with bright fluorescent proteins (Pan et al., 2013; Li et al., 2019; Balla et al., 2020). However, this method is work-intensive and bears the risk of introducing artifacts by expression of exogenous proteins (Lipták et al., 2019). Spectral overlap of fluorescent proteins typically prevents to study more than 3–5 biomarkers at once. Moreover, out-of-focus photobleaching, toxicity, scattering, absorption, and signal loss are common drawbacks when imaging biological tissue with single photon excitation (Georgakoudi and Quinn, 2012). Multiphoton techniques can be used to overcome these restrictions. Compared to single photon fluorescence microscopy multiphoton improves depth penetration and reduces photo damage as a result of employing near infrared femtosecond lasers. It is uniquely suited to perform studies with "minimal" invasion over long periods of time to provide excellent insight on dynamic biological processes offering time scales from microseconds to days or weeks.

Multiphoton microscopy based on nonlinear optical processes is the method of choice for imaging living, intact tissue on a molecular level throughout the entire organism with molecular endogenous and exogenous contrast. Two-photon excited fluorescence (TPEF) and second harmonic generation (SHG) imaging have been used for imaging zebrafish (Abu-Siniyeh and Al-Zyoud, 2020). Neural population activity and organogenesis have been investigated in transgenic fluorescent zebrafish with TPEF microscopy (Renninger and Orger, 2013; Abu-Siniyeh et al., 2016). Gene expression and wound healing have been studied using endogenous contrast mechanism by means of SHG (Hsieh et al., 2008; LeBert et al., 2015, 2016). Still, metabolic adaptations are challenging to image in the native cell environment, e.g., without the use of labels, and few optical methods are available. TPEF has the capability to image metabolic activity but is limited to the cellular optical redox ratio by inducing the endogenous fluorescence response of flavin adenine dinucleotide (FAD) and the reduced form of nicotinamide adenine dinucleotide (NADH) and detecting either the fluorescence intensity or fluorescence lifetime (FLIM) (Skala et al., 2007; Quinn et al., 2013; Stringari et al., 2017; Cao et al., 2020).

Coherent Raman scattering (CRS) has emerged as powerful imaging tool offering a unique opportunity to image metabolism by tracking specific endogenous molecules of interest (Yue and Cheng, 2016). CRS is able to excite molecular vibrations offering label-free chemically selective imaging, because certain molecules have a distinctive signature produced by the vibrations of their chemical bonds (Cheng and Xie, 2016). CRS is a nonlinear optical process driving a vibrational transition in a molecule with two photons, followed by a third photon that probes the induced vibrational coherence of this molecule. Two short pulsed laser beams, the pump of frequency *ω*_*p*_ and the Stokes of frequency *ω*_*S*_, are used. When the frequency beating *ω*_*p*_ − *ω*_*S*_ matches the vibrational frequency of a molecule, two major processes occur simultaneously, namely stimulated Raman scattering (SRS) and coherent anti-Stokes Raman scattering (CARS). In SRS, the signal is obtained by an increased intensity on the Stokes beam (stimulated Raman gain) or a decreased intensity on the pump beam (stimulated Raman loss), and thus SRS requires modulation schemes and lock-in or tuned amplifier detection approaches (Freudiger et al., 2008; Slipchenko et al., 2012). CARS signal is generated at a new frequency 2*ω*_*p*_ − *ω*_*S*_, away from the excitation frequency and thus the experimental requirements are more relaxed in respect to SRS, e.g., the signal can be detected with a photomultiplier tube (PMT). CRS allows the use of the spectral focusing method to add a frequency dimension to the microscopic image allowing for fast and easy switching of the vibrational excitation frequency by means of chirped laser pulses (Hellerer et al., 2004). Through the obtained hyperspectral image spatial discrimination of different molecular components within the sample can be performed. CRS has been extensively used to visualize lipids through their carbon-hydrogen (C-H) bonds due to their high Raman cross-section (Huff et al., 2008; Kim et al., 2010; Le et al., 2010b). Several studies showed that CRS microscopy can be used to investigate different types of living tissues including cell culture (Potcoava et al., 2014), mouse (Le et al., 2009; Uckermann et al., 2014; Liu et al., 2019), *C. elegans* (Le et al., 2010a; Shi et al., 2018), and zebrafish (den Broeder et al., 2017) models. As mentioned before, altered lipid metabolism has been identified as a biomarker for several types of tumors and thus CRS represents a promising way to investigate lipid content within the tumor microenvironment (TME).

The ability to describe subtle changes in tumor cells and their environment is an important step to understand molecular dynamics. Conventionally, statistical imaging analysis methods based on the intensity of individual pixels, namely mean and standard deviation, are used to extract quantitative values. In recent years, radiomics has emerged as scientific field that extracts large amounts of quantitative features from images to characterize imaging patterns, conventionally, from radiological and hybrid datasets (Lambin et al., 2012). The radiomic process can be divided into distinct steps, such as image acquisition and reconstruction, image segmentation, features extraction and qualification, analysis, and model building. Each step needs careful evaluation for the construction of robust and reliable models to be transferred into clinical practice for the purposes of diagnosis, prognosis, non-invasive disease tracking, and evaluation of disease response to treatment (Rizzo et al., 2018; Devkota et al., 2020).

In this paper, we performed hyperspectral CARS microscopy to investigate lipid metabolism in a zebrafish cancer model. The CARS microscope, based on a compact and cost effective femtosecond Ti:sapphire laser with an inherently synchronized high power Yb fiber amplifier for Stokes generation as described in Andreana et al. (2017), was used to analyze the lipid content in oncogene expressing and healthy zebrafish larvae *in vivo*. Radiomics features were used to distinguish between healthy and cancerous tissue based on the lipid content shown on the CARS images. We followed the larvae longitudinally from 72 h-post-fertilization (hpf) up to 120 hpf and monitored changes that occur in the lipid content of an emerging tumor.

## 2. Materials and Methods

### 2.1. Animal Model

**Figure 1:**
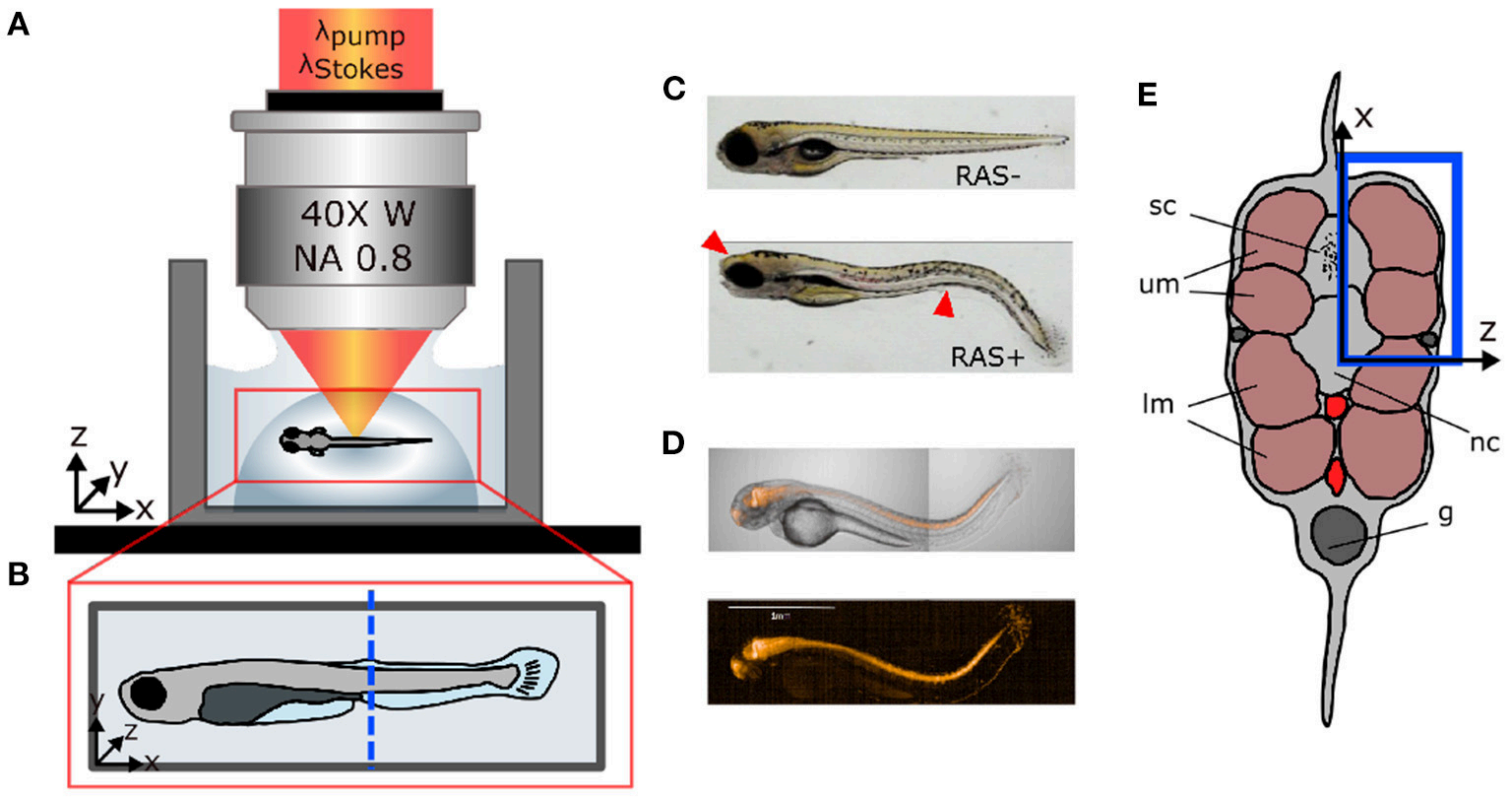
Sample preparation for *in vivo* imaging. **(A)** Lateral view of coherent anti-Stokes Raman scattering (CARS) microscope objective showing the fish mounted in agarose gel. **(B)** Top view of fish mount showing the imaging region of interest (ROI) (blue dashed line). **(C)** Microscopic white light images of RAS- and RAS+ larvae showing the cancer phenotype (red arrows). **(D)** mCherry expression at location of RasG12V expression in a 96 hpf larvae. **(E)** Cross-section of the zebrafish larvae showing the ROI in depth (blue rectangle) with: sc, spinal cord; um, dorsal myotomes; lm, lower myotomes; nc, notochord; g, gut.

Zebrafish (*D. rerio*) were maintained at standard conditions (Kimmel et al., 1995; Westerfield, 2000) according to the guidelines of the local authorities under licenses GZ:565304/2014/6 and GZ:534619/2014/4. Transgenic zebrafish strains *Et(SP8b:KalTA4,UAS:mCherry) Tg(UAS:EGFP-Hsa.HRAS_G12V)io6* were used to target oncogene expression to the central nervous system (Distel et al., 2009; Santoriello et al., 2010). This leads to hyperproliferation of neural cells within the brain and the spinal cord in zebrafish larvae and, causing prominent “curved tail” and head deformation phenotypes at 5 days after fertilization (Figure 1C). To give an overview of the fluorescent co-expression with the oncogene, we show mCherry expression at location of RASG12V in Figure 1D. The image was taken on the PerkinElmer Operetta CLS with 5x Air objective. From now on, we will refer to oncogene-expressing and healthy larvae as “RAS+” and “RAS-” to indicate the presence or the absence of oncogenic RAS expression, respectively.

### 2.2. Sample Preparation

After spawning, eggs from RAS+ and RAS- zebrafish were maintained in E3 medium at 28°C under standard conditions. To suppress pigmentation, 1-phenyl 2-thiourea (PTU, CAS Number:103-85-5; Sigma Aldrich) was added to the medium at 24 hpf. For imaging, zebrafish were manually dechorionated and larvae showing a healthy or strong RAS-mediated phenotype were selected and transferred to a small water tank. Before imaging, zebrafish larvae were anesthetized in 100 mg/L tricaine (CAS Number 886-86-2; Sigma-Aldrich). Subsequently, larvae were transferred into a pipette with 250 *μ*L of 1 % ultra-low gelling temperature agarose at 32°C (CAS Number 9012-36-6; Sigma Aldrich). The volume containing the zebrafish larvae was then released into a sterilized glass bottom dish forming a semi-spherical volume (Figure 1A) to fix the larvae in space for imaging. The larvae were manually micro-positioned sideward, perpendicular to the optical axis and away from gel borders under a microscope as shown in Figures 1A,B. After gelification, the glass bottom dish was filled with E3 medium to preserve the humidity level of the gel during the imaging session and ensure survival of larvae. At this point, larvae were ready to be imaged. CARS images were taken from the same region of interest (ROI) in the tail for all larvae at the position of the two dorsal myotomes (blue rectangle in Figure 1E). The end of the yolk extension served as morphological reference (blue dashed line in Figure 1B). This covers the region, where strong hyperproliferation of neural spinal cord cells can be observed in RAS+ larvae.

### 2.3. Nile Red Fluorescence Imaging

Nile red (CAS Number 7385-67-3; Thermo Fisher Scientific) fluorescence imaging has been used to countercheck the signal of the label-free contrast by means of CARS microscopy. Indeed, its potential as fluorescent lipid stain has been exploited for many years (Greenspan et al., 1985). It is highly sensitive to the microenvironment polarity that induces distinct chromatic properties on the fluorescence excitation/emission spectra. The fluorescent peak emission is blue shifted as the polarity of the environment decreases, from deep red for polar lipids to yellow for neutral lipids. Therefore, it has been extensively used to selectively investigate polar and neutral lipids such as phospholipid bilayer and triglyceride. In this work, we make use of this chromatic property to image triglyceride content in living zebrafish larvae. *In vivo* imaging of 120 hpf zebrafish larvae stained with Nile red, following the protocol as in Minchin and Rawls (2011), has been performed with a Leica confocal microscope using 510 nm laser line excitation and detection window between 520 and 605 nm to ensure visualization of triglycerides' tissue distribution content. For comparison reasons, we have used a Fluotar VISIR 25x/0.95 water immersion objective, which gives a rather similar spatial resolution to the CARS images.

### 2.4. Imaging Conditions and System

*In vivo* zebrafish measurements were conducted using an epi-detecting label-free imaging platform as described in Andreana et al. (2017) and the geometry shown in Figures 1A–C. In brief, CARS imaging was performed by means of chirped pulses, the so-called spectral focusing implementation, with the pump and the Stokes laser beams centered at 805 and 1,050 nm, respectively, not only to be in resonance with but also covering the C-H stretch vibration region. Spectral focusing was configured to reach a spectral resolution of ~ 35 cm^−1^, enabling sufficient spatial-spectral discrimination between the CH_2_ stretching vibration at 2,845 cm^−1^ corresponding to lipid molecules and contribution from the agarose gel at ~ 3000 cm^−1^. The laser beams were focused onto the sample through a 40x/0.8 water immersion microscope objective with a 0.8 numerical aperture and a working distance of 3.5 mm. Pump and Stokes beam powers at the sample were kept constant during all the experiments at 25 mW and 10 mW, respectively. Epi-CARS signal was detected through the same illuminating objective spectrally separated from the excitation beams and detected with a PMT. The combination of spectral focusing CARS and epi-detection scheme ensures an improved ratio between resonant and nonresonant contributions of the CARS signal. Average acquisition time was 0.8 s for a 512 × 512 pixel CARS image. The spectral scan data set of 240 time points took about 100 s over the C-H stretch region from 2,500 to 3,500 cm^−1^.

### 2.5. Data Analysis

While the ability to track associations between lipid CARS images and pathology is important, it is equally important to have the ability to track such correlations using quantifiable measures for objective comparison. Image data analysis was performed on the CARS images to highlight these associations.

**Figure 2:**
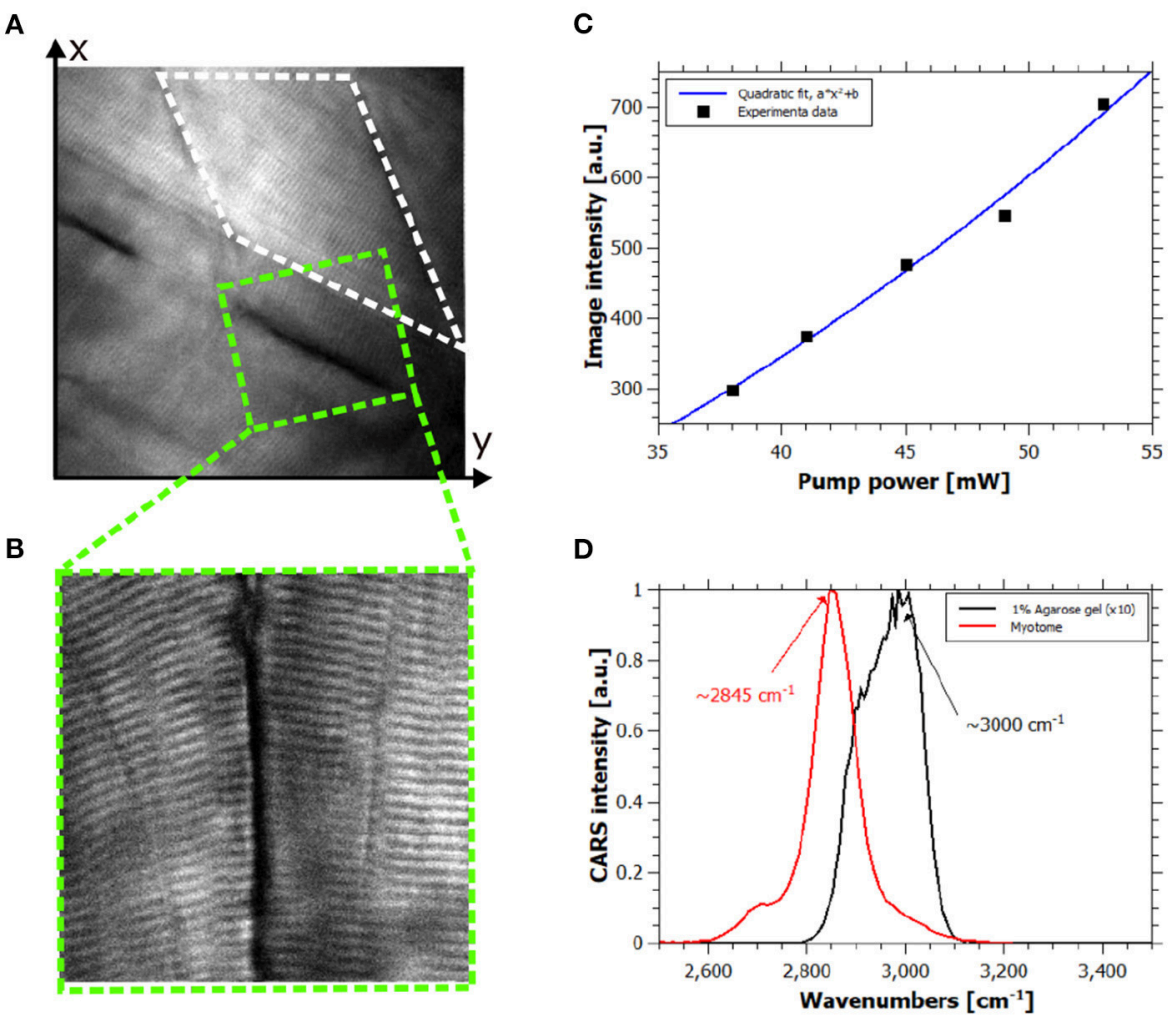
*In vivo* hyperspectral coherent anti-Stokes Raman scattering (CARS) images. **(A)** CARS image of healthy zebrafish larva at 120 hpf at 2,845 cm^−1^, field of view (FOV) is 80 × 80 *μ*m^2^. **(B)** High-resolution CARS image of the green dashed square in **(A)** showing contrast coming from the lipid content of the muscle cells, FOV is 30 × 30 *μ*m^2^. **(C)** Quadratic power dependency of the CARS signal of the white dashed rectangle area in **(A)** respect to the pump power intensity. R-squared of the quadratic fit is 0.9889. **(D)** Spectral information provided by hyperspectral CARS allowing discrimination between the resonant signal of lipids and 1% agarose gel.

Initially, a simple evaluation of the CARS images properties was performed based on the intensity of individual pixels to relate the amount of lipid deposited in a specific ROI with the image contrast. Other than the absolute intensity value detected by the sensor (which will later become the image's pixel), we used mean and standard deviation to obtain the intensity levels of the selected ROI in the images. The mean provides measures of the overall lightness/darkness of the ROI, while the standard deviation describes its overall contrast. These measures are associated to the amount of lipid detected in a specific region. The identified ROI for these measurements corresponded to a single dorsal myotome of the zebrafish larvae (white dashed line in Figure 2A). For comparative measurements between RAS+ and RAS- larvae at progressing developmental stages, the ROIs were matched in terms of dimensions (number of pixels). Image intensity of the ROI was normalized as a function of the PMT gain and thresholding was performed taking the notochord wall intensity as a reference. Thereby, we ensured that the measurement was not affected by slightly different experimental settings for each larvae. Mean and standard deviations were calculated by using the open source software ImageJ (Schindelin et al., 2012). At this point, we performed statistical analysis for each developmental stage between RAS+ and RAS- zebrafish larvae (n = 3) on the normalized ROI. Shapiro–Wilk W test for normality distribution was initially performed (Royston, 1992). The null hypothesis at significance level of 0.05 was in all 3 cases (76, 96, and 120 hpf) not rejected. Furthermore, two-tailed Welch's *t*-test was used, thereby *p*-values smaller than 0.05 were considered as significantly different.

Moreover, exploratory quantitative analysis of the underlying image pattern characteristics was performed including numerical radiomic features. Hence, the CARS images underwent radiomic analysis utilizing the radiomic engine of python version 3.6.8 (pyradiomics version 2.2.0, van Griethuysen et al., 2017), which has been certified as being compliant with the Imaging Biomarker Standardization Initiative (IBSI) guidelines (Zwanenburg et al., 2020). The radiomic features were correlated with their respective label (RAS-/RAS+) using point-biserial correlation (Tate, 1954). The bin size for pixel value discretization before radiomics was chosen as 98 from the value range of 1–100 automatically so that the lowest feature significance *p*-value was minimized. This bin width resulted in max 5 number of bins. Features having *p*-value lower than 0.05 were further analyzed and decoded to describe the textural characteristics differences of the RAS+ and RAS- cases.

## 3. Results

In order to confirm the origin of the CARS signal from all measurements, we performed experimental checks on the collected signals. Typically, the contrast in CARS microscopy arises from molecular specific and non-specific signals, the so-called resonant and nonresonant contributions. Indeed, in this complex living system this is a key point for the correct interpretation of the images. Considering the representative image in Figure 2A and the corresponding high-resolution image in Figure 2B, the power dependency and the spectral shape of the signal of the myotome (white dashed rectangle) and the sarcomeres were evaluated. Indeed, quadratic and linear dependencies of the signal were found for pump and Stokes intensities (see Figure 2C), respectively, and the detected signal disappeared when pump or Stokes beam was out of resonance or blocked. Hence, the collected signal was assigned as resonant CARS contribution of muscle fibers. Moreover, the spectral information carried by each pixel by means of spectral focusing CARS allowed the discrimination of the resonant molecules. We assigned the signal at 2845 cm^−1^ to the lipid-rich structure of the muscles fibers as shown in Figure 2D. We also spectrally identified and characterized the resonant signal of the agarose gel, which did not interfere with the resonant lipid signal of interest as shown in Figure 2D.

**Figure 3:**
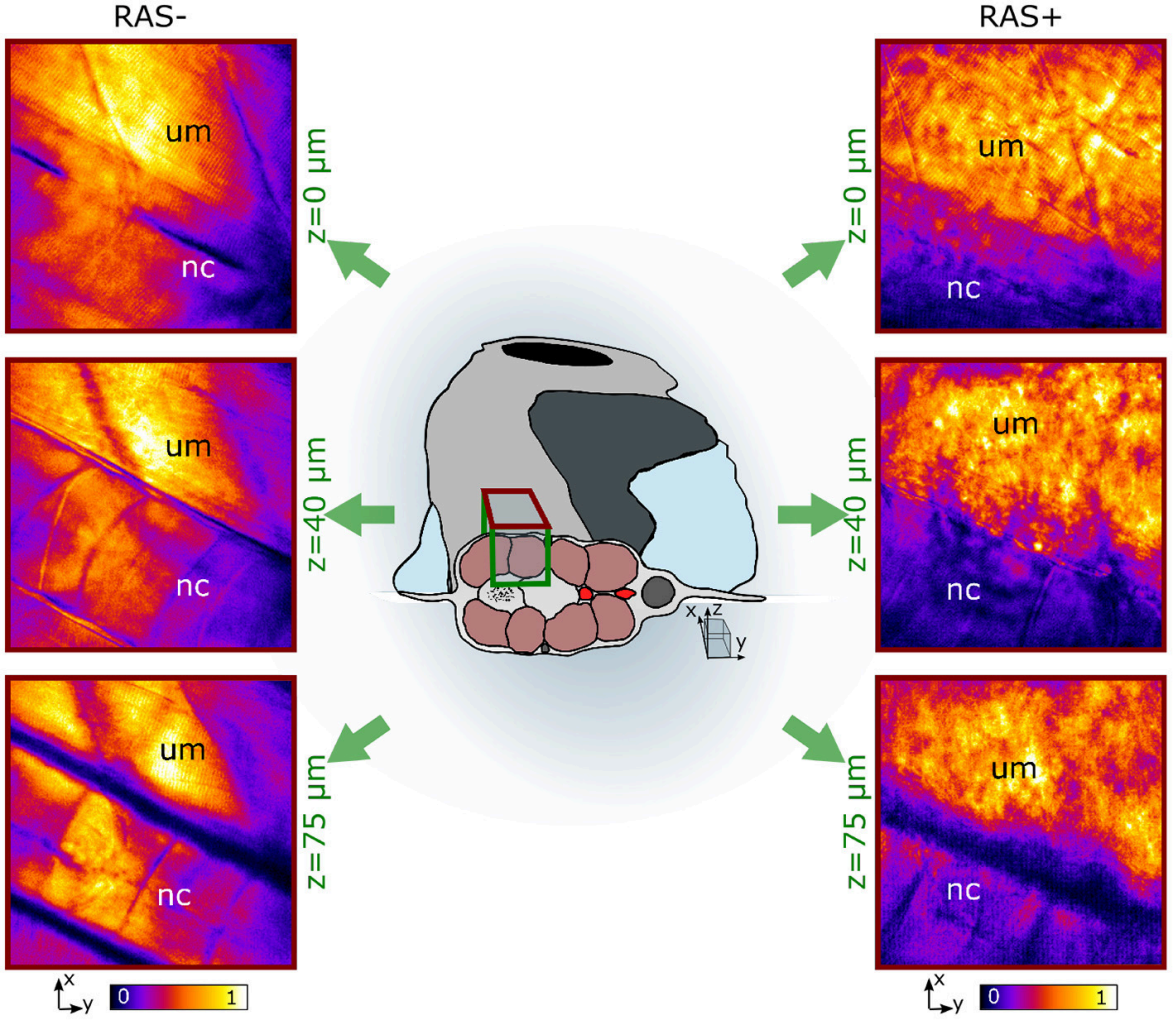
*In vivo* coherent anti-Stokes Raman scattering (CARS) images at 2,845 cm^−1^ at three different depths for RAS- (left) and RAS+ (right) larvae. nc, notochord; um, dorsal myotome region. All images have a FOV of 100 × 100 *μ*m^2^.

For careful investigation of the lipid distribution in the CARS images and its role during cancer formation, we performed two different measurements. We first analyzed the lipid distribution of RAS- and RAS+ larvae in depth along the z direction on the selected ROI as described in section 2.2 from the muscle surface down to the middle of the notochord (Figure 3). Subsequently, we investigated the behavior over time from 72 to 120 hpf. For comparative investigation, we focused the laser beams always on the same ROI and at a depth of around 50 μm from the muscle surface.

Figure 3 shows representative images of the lipid distribution in depth for 120 hpf larvae. In healthy larvae, the well-organized lipid distribution is clearly visible. The corresponding CARS signals are coming from lipid-rich structures within the dorsal myotome (um), where muscle fibers are clearly visible. The myotomes are followed in depth through their lipid content. The notochord (nc) is also visible at depths from 40 to 75 *μ*m since its cell membrane structure is composed of a mixture of lipids and proteins. The CARS images of RAS+ fish have a totally different appearance. The organized lipid distribution from the muscle fibers found in healthy larvae can be barely identified at the surface. Deeper in the tissue this arrangement is completely lost and a cluster of high-intensity lipid signal is visible. We can identify a region, where there is high-clustered lipid content overwhelming the expected CARS signal coming from the muscle fibers at depths from 40 to 75 *μ*m as observed in the healthy case. This ROI corresponds to the enlarged spinal cord location, where RAS expressing cells are situated and are proliferating.

**Figure 4:**
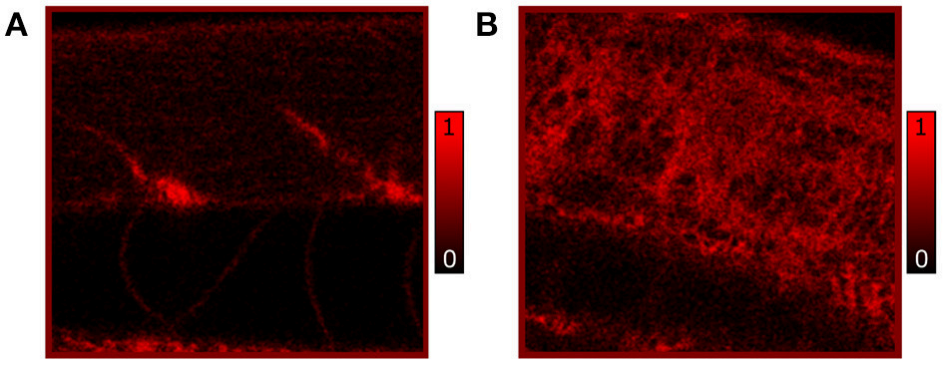
*In vivo* confocal images of Nile Red stained RAS- **(A)** and RAS+ **(B)** 120 hpf larvae imaged with 25x, 0.95 NA water immersion objective. The contrast is based on fluorescence of Nile Red excited with 510 nm laser excitation showing lipid (triglycerides) distribution and content. All images have a field of view (FOV) of 100 × 100 *μ*m^2^.

Additionally, we performed independent *in vivo* measurements to confirm the distribution of lipids as observed by CARS microscopy. Here, Nile Red staining highlighting neutral lipids (triglycerides) in RAS+ and RAS- larvae was performed and 120 hpf larvae were imaged by confocal microscopy to show triglycerides content. The comparative results of Nile red showing similar phentotype than the CARS images are shown in Figure 4.

**Figure 5:**
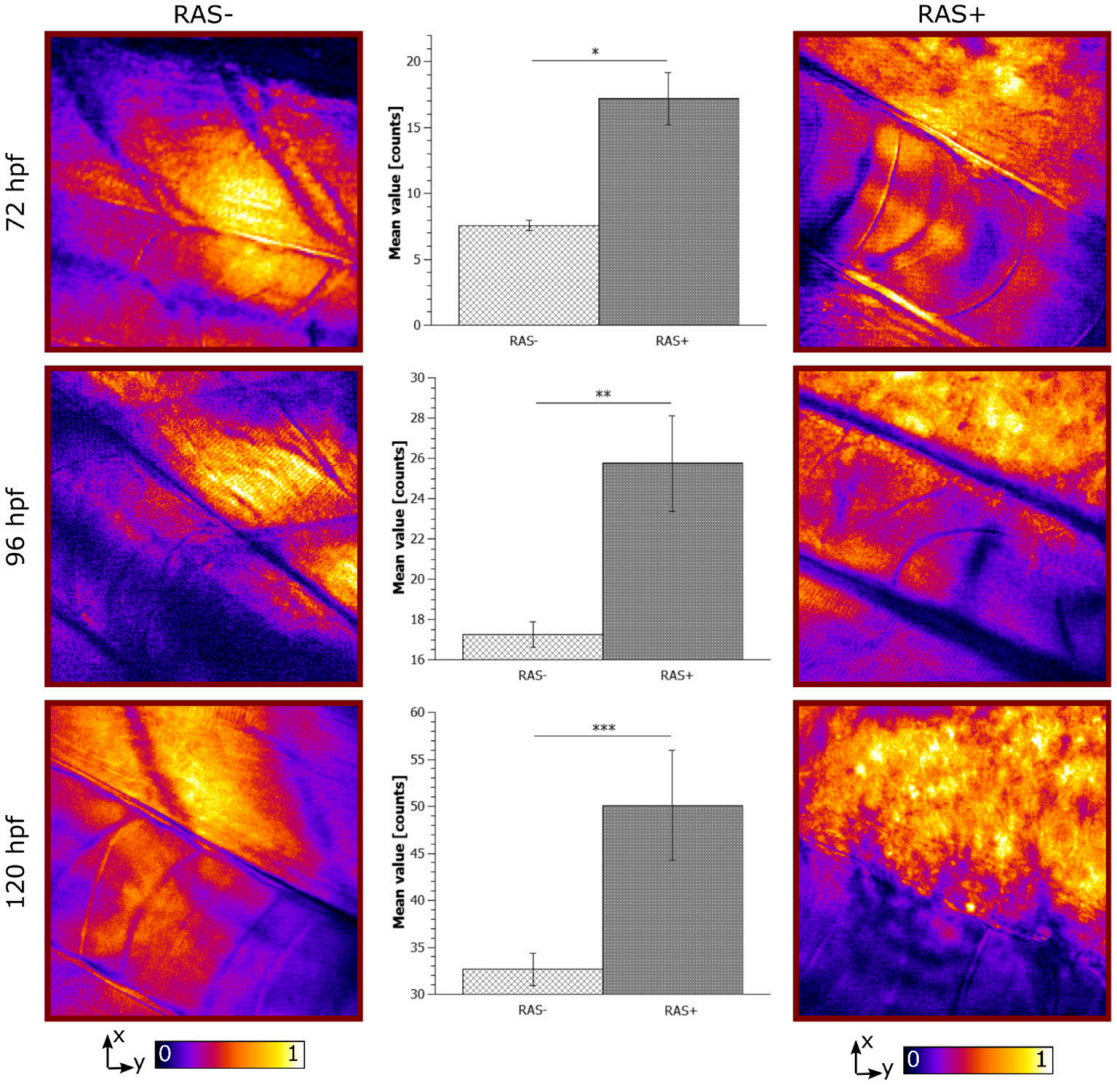
Longitudinal *in vivo* coherent anti-Stokes Raman scattering (CARS) images at 2,845 cm^−1^ for RAS- and RAS+ larvae from 72 to 120 hpf (left and right columns). All images have a field of view (FOV) of 100 × 100 *μ*m^2^. The single image contrast is optimized for better visualization. Center column shows the lipid distribution on the specific region of interest (ROI). Significant differences between the 3 groups with *n* = 3 (72, 96, and 120 hpf) are shown (^*^p < 0.02, ^**^p < 0.03, and ^***^p < 0.04).

The CARS images from the longitudinal study from 72 to 120 hpf are shown in Figure 5. RAS- larvae at different developmental stages show well-organized myotomes and muscle fibers at all time points. The corresponding CARS signals increase according to the growth of the larvae due to the augmented lipid content in muscle fibers. In the RAS+ fish, the well-organized myotome structure is not visible anymore at any stages as predicted from results with 120 hpf old fish. An increasing clustered lipid content is observed. As well the expected contrast coming from the notochord is not as clear as in the healthy cases due to the high lipid content overwhelming all other signals. Indeed, the dynamic range of the images, especially for the 120 hpf cases, has been mainly filled with the lipid signal coming from the ROI corresponding to the dorsal myotomes. Hence, the lipid signal of the notochord is not visible anymore. At 72, 96, and 120 hpf, the clustered detected lipid signals correspond to the ROI, where hyperproliferating cells are situated.

## 4. Discussion

The observed lipid content in the CARS images has been investigated by means of mean and standard deviation of the intensity distribution within a single image and radiomic analysis as described in section 2.5. First, mean and standard deviations on a normalized ROI have been calculated. Results are highlighted in Figure 5 for 72, 96, and 120 hpf RAS+ and RAS- larvae. When comparing results at the three developmental time points for the healthy cases only, the mean value indicates that lipid content is following the growth of the myotome as an increase in lipid signal can be observed. Still, the same trend is also clearly visible for the RAS+ cases. However, clear evidence of different lipid content is observable when comparing RAS- to RAS+ larvae at each of the three analyzed time points. Since the mean value provides a measure of the overall contrast of the CARS images in the selected ROI and the image contrast is related to the amount of lipids, it can be seen that the region with oncogene expressing cells in the RAS+ larvae contain higher amounts of lipids compared to healthy larvae at the same time point. The standard deviation provides a confirmation that a different biological mechanism is taking place involving lipid molecules. Indeed, the high standard deviation as observed in the RAS+ larvae yields to high clustering in the images as shown in Figures 3, 5, and thus high variation in the lipid distribution within the ROI. The above-described pattern is supported by the Nile Red staining experiments. Indeed, after performing the same image analysis based on pixel intensity, confocal images in Figure 4 show ~ 1.6 times higher lipid content in the RAS+ larvae when compared with the RAS- one.

Second, for objective evaluation radiomics analysis has been performed. Dependence variance is a gray level dependence matrix (GLDM) metrics, which characterizes the gray value dependence size in the image. (i) Dependence is defined as the number of connected pixels that are dependent (similar) to a center pixel, meaning in case of more homogeneous textures this dependency is higher. (ii) Normalized non-uniformity measures the similarity of run lengths in the image. (iii) Run length refers to the number of consecutive pixels having the same values in the image. Lower non-uniformity value indicates that the run length similarities are lower, which corresponds to more heterogeneous patterns. Short run emphasis describes the distribution of short run lengths. Higher short run emphasis value indicates more fine textures. These three identified significant radiomic features had a negative correlation coefficient, implying that in case of RAS+ larvae these values are all decreased compared to RAS- larvae. This finding correlates with visual assessment, where RAS- cases represent a more balanced pattern, composed of small but homogeneous subregions. Overall, these findings indicate that the lower the dependence variance (surrounding pixels are different than center pixel), the lower the run length similarities (high variation in homogeneous subgroup sizes), and the lower the short run length emphasis (many small homogeneous connected pixels – distorted texture), the more likely a tissue is malignant. For the distribution and correlation of these prominent features, see Table 1. These initial findings imply that the textural characteristics of RAS+ vs. RAS- cases can be more accurately characterized with advanced radiomic analysis compared to more conventional statistical tools, e.g., mean and variance. This could open the potential for future radiomic-based predictive models that aim to automatically characterize the aggressiveness of tumors in zebrafish specimen.

**Table 1:** Distribution of the three most prominent features associated with RAS+ vs. RAS- for 72, 96, and 120 hpf zebrafish larvae.

**Feature**	***p*-value**	**R(correlation coefficient)**
Dependence Variance (GLDM)	0.020	–0.882
RunLengthNonUniformityNormalized (GLRLM)	0.041	–0.830
ShortRunEmphasis (GLRLM)	0.042	–0.827

*SRE, short run emphasis; DV, difference variance; RLNUN, normalized non-uniformity (run-length matrix). The correlation coefficient R indicates the relationship of the continuous radiomic features generated from the images at each time point and the binary RAS- vs. RAS+ variable*.

The results shown give an objective evaluation that hyperproliferative cells accumulate higher amounts of lipids compared to untransformed cells in our animal model from early stages on. Indeed, under non-pathological cellular conditions there is a balance between lipid production and the amount used. Recent studies have suggested that cancer cells with RAS mutations exhibit altered metabolism including an enhanced lipid synthesis (Oba et al., 2018). The high lipid accumulation observed by means of CARS microscopy in RAS+ larvae fits with these previous studies. Changes in lipid content as observed in our longitudinal study indicate that lipid metabolism alterations could serve as biomarker that can be traced over time by non-invasive and label-free CARS microscopy in combination with radiomics opening up clinical applications (Figure 5).

## 5. Conclusions

Label-free *in vivo* imaging of lipids in zebrafish larvae from 72 to 120 hpf has been performed by means of hyperspectral CARS microscopy. Lipid distribution was investigated in oncogene-expressing and healthy larvae by means of standard statistical tools and more advanced tools like radiomics. It was shown that multiple radiomic features have a significant negative correlation with the cancer state of zebrafish larvae. However, due to the small sample size for the radiomic analysis, further investigation with a larger cohort is needed in order to determine whether the discovered relation holds true in a more representative cohort. It has been demonstrated that CARS microscopy can be used to track lipid content in living larvae revealing increased lipids in tumor-developing zebrafish larvae. This trend has been confirmed by Nile Red staining experiments. Our results indicate that CARS microscopy is a suitable method to investigate lipid metabolism in living zebrafish larvae during development and tumor formation. We anticipate that using hyperspectral CARS microscopy will allow for detailed characterization of lipid metabolism alterations during tumorigenesis. Furthermore, we envision that our CARS microscopy approach can be implemented in label-free high throughput via radiomics automated screening platforms to identify compounds with anti-tumor effects by targeting lipid metabolism in zebrafish, which could ultimately be translated to clinical applications. Our platform could help to better understand mechanisms underlying cancer development. While the data and the analysis presented in this paper are preliminary and further investigation with a larger number of samples is required, our proposed approach based on the combination of radiomic analysis and label-free CARS microscopy has high potential for an improved understanding of lipid metabolism and for high throughput *in vivo* zebrafish drug screening.

## Data Availability Statement

The original contributions presented in the study are included in the article/supplementary material, further inquiries can be directed to the corresponding author/s.

## Ethics Statement

Ethical review and approval was not required for the animal study because in this study zebrafish larvae from 72 to 120 h-post-fertilization have been investigated which do not require any ethic approval.

## Author Contributions

MA and AU collected and analyzed the CARS data. CS collected and analyzed the Nile red data. CPS and LP performed the radiomic feature analysis. CS and MD generated the zebrafish model and provided the zebrafish larva. MA, AU, CS, and MD wrote the manuscript. RL, WD, MD, and AU coordinated and supervised the experiments. All authors approved the final manuscript.

## Conflict of Interest

The authors declare that the research was conducted in the absence of any commercial or financial relationships that could be construed as a potential conflict of interest.
