# Erratum: Toward Quantitative *in vivo* Label-Free Tracking of Lipid Distribution in a Zebrafish Cancer Model

**DOI:** 10.3389/fcell.2021.746196

**Published:** 2021-08-11

**Authors:** Frontiers Production Office

**Affiliations:** Frontiers Media SA, Lausanne, Switzerland

**Keywords:** zebrafish, cancer model, lipid metabolism, label-free microscopy, coherent anti-Stokes Raman scattering

Due to a typesetting error, the names of the authors in a reference were misspelled. Instead of “Lipt” and “Bsze” it should be “Lipták” and “Bősze”. The full reference should be:

Lipták, N., Bősze, Z., and Hiripi, L. (2019). GFP transgenic animals in biomedical research: a review of potential disadvantages. *Physiol. Res*. 68, 525–530. doi: 10.33549/physiolres.934227

The publisher apologizes for this mistake. The original article has been updated.

